# Supplementary material for: SyNDI: synchronous network data integration framework
Source: BMC Bioinformatics. 2018 Nov 6;19:403. doi: 10.1186/s12859-018-2426-5 (PMC6219086; doi:10.1186/s12859-018-2426-5)
Supplement: Supplementary file 5 — Files of the example presented in Additional file 4. This additional file is a zip package that contains all files used in Additional file 4. Detailed descriptions of each of these files are presented in a README file included in this zip package. (GZ 30411 kb) [file 12859_2018_2426_MOESM5_ESM.gz › SyNDITools/Meme2Fimo/meme_4.9.0_4.tar.gz!/meme_4.9.0/doc/lipo.meme.html]

MEME


|  |  |  |  |  |  |
| --- | --- | --- | --- | --- | --- |
| **Command line** **Training Set** **First Motif** **Summary of Motifs** **Termination** **Explanation** | | | | | |

  


### --- MEME - Motif discovery tool ---

MEME version 3.0 (Release date: 2001/03/03 12:02:21)

For further information on how to interpret these results or to
get a copy of the MEME software please access http://meme.sdsc.edu.

This file may be used as input to the MAST algorithm for
searching sequence databases for matches to groups of motifs. MAST is
available for interactive use and downloading at http://meme.sdsc.edu.

### --- REFERENCE ---

If you use this program in your research, please cite:

Timothy L. Bailey and Charles Elkan, "Fitting a mixture model by
expectation maximization to discover motifs in biopolymers", Proceedings
of the Second International Conference on Intelligent Systems for
Molecular Biology, pp. 28-36, AAAI Press, Menlo Park, California, 1994.

### --- TRAINING SET ---

```
DATAFILE= lipocalin.s
ALPHABET= ACDEFGHIKLMNPQRSTVWY
Sequence name           Weight Length  Sequence name           Weight Length  
-------------           ------ ------  -------------           ------ ------  
ICYA_MANSE              1.0000    189  LACB_BOVIN              1.0000    178  
BBP_PIEBR               1.0000    173  RETB_BOVIN              1.0000    183  
MUP2_MOUSE              1.0000    180
```

  


### --- COMMAND LINE SUMMARY ---

```
This information can also be useful in the event you wish to report a
problem with the MEME software.

command: meme lipocalin.s -mod oops -protein -nmotifs 3 

model:  mod=          oops    nmotifs=         3    evt=           inf
object function=  E-value of product of p-values
width:  minw=            8    maxw=           50    minic=        0.00
width:  wg=             11    ws=              1    endgaps=       yes
nsites: minsites=        5    maxsites=        5    wnsites=       0.8
theta:  prob=            1    spmap=         pam    spfuzz=        120
em:     prior=        dmix    b=               0    maxiter=        50
        distance=    1e-05
data:   n=             903    N=               5

sample: seed=            0    seqfrac=         1
Dirichlet mixture priors file: prior30.plib
Letter frequencies in dataset:
A 0.072 C 0.029 D 0.069 E 0.078 F 0.043 G 0.058 H 0.025 I 0.048 K 0.086 
L 0.087 M 0.018 N 0.053 P 0.032 Q 0.029 R 0.031 S 0.059 T 0.048 V 0.070 
W 0.017 Y 0.050 
Background letter frequencies (from dataset with add-one prior applied):
A 0.072 C 0.029 D 0.068 E 0.077 F 0.043 G 0.057 H 0.026 I 0.048 K 0.086 
L 0.087 M 0.018 N 0.053 P 0.033 Q 0.029 R 0.031 S 0.059 T 0.048 V 0.069 
W 0.017 Y 0.050
```

### --- | | | | --- | --- | | **P** **N** | | MOTIF 1     width = 19     sites = 5     llr = 187     E-value = 8.9e-006 ---

|  |  |  |  |  |  |  |  |  |  |  |  |  |  |  |  |  |  |  |  |  |  |  |  |  |  |  |  |  |  |
| --- | --- | --- | --- | --- | --- | --- | --- | --- | --- | --- | --- | --- | --- | --- | --- | --- | --- | --- | --- | --- | --- | --- | --- | --- | --- | --- | --- | --- | --- |
| Simplified A : : : : : : : 2 2 : 6 : 2 : : 2 : 8 : | | | | | | | | | | | | | | | | | | | | | | | | | | | | | |
| pos.-specific C : : : : : : : : : : : : : : : : : : : | | | | | | | | | | | | | | | | | | | | | | | | | | | | | |
| probability D : : 2 2 : 8 : : : : : : : : : : : : : | | | | | | | | | | | | | | | | | | | | | | | | | | | | | |
| matrix E : : 2 : : : : 2 : : : : 2 : : 4 : : : | | | | | | | | | | | | | | | | | | | | | | | | | | | | | |
| F : : : : 8 : : : : 4 : : : : : : : : : | | | | | | | | | | | | | | | | | | | | | | | | | | | | | |
| G : 2 : 2 : : : : : : : a : : : : : : : | | | | | | | | | | | | | | | | | | | | | | | | | | | | | |
| H : : : : : : : : : : 2 : : : 4 : : : : | | | | | | | | | | | | | | | | | | | | | | | | | | | | | |
| I : : : : : : 2 : : 2 : : : : : : 4 2 : | | | | | | | | | | | | | | | | | | | | | | | | | | | | | |
| K : 2 2 : : : 2 : 4 : : : 2 : : : : : 6 | | | | | | | | | | | | | | | | | | | | | | | | | | | | | |
| L : : : : 2 : 2 : : : : : : : : : 2 : 2 | | | | | | | | | | | | | | | | | | | | | | | | | | | | | |
| M : 2 : : : : : : : : : : : : : : 2 : 2 | | | | | | | | | | | | | | | | | | | | | | | | | | | | | |
| N : : 2 6 : 2 : : 2 : 2 : : : : : : : : | | | | | | | | | | | | | | | | | | | | | | | | | | | | | |
| P 4 : : : : : : : : : : : : : : : : : : | | | | | | | | | | | | | | | | | | | | | | | | | | | | | |
| Q : : : : : : : 2 : : : : : : : : : : : | | | | | | | | | | | | | | | | | | | | | | | | | | | | | |
| R : : 2 : : : : : 2 : : : : : : : : : : | | | | | | | | | | | | | | | | | | | | | | | | | | | | | |
| S : : : : : : : 4 : : : : : : : 2 : : : | | | | | | | | | | | | | | | | | | | | | | | | | | | | | |
| T 4 : : : : : : : : : : : 4 : : 2 : : : | | | | | | | | | | | | | | | | | | | | | | | | | | | | | |
| V 2 4 : : : : 2 : : 2 : : : : : : 2 : : | | | | | | | | | | | | | | | | | | | | | | | | | | | | | |
| W : : : : : : 2 : : : : : : a 2 : : : : | | | | | | | | | | | | | | | | | | | | | | | | | | | | | |
| Y : : : : : : : : : 2 : : : : 4 : : : : | | | | | | | | | | | | | | | | | | | | | | | | | | | | | |
| .\|  \|  \|  \|  \|  \|  \|  \|  \|  \|  \|  \|  \|  \|  \|  \|  \|  \|  \|  \|  \|  \|  \|  \|  \|  \|  \|  \|  \|  \|  \| \| --- \| --- \| --- \| --- \| --- \| --- \| --- \| --- \| --- \| --- \| --- \| --- \| --- \| --- \| --- \| --- \| --- \| --- \| --- \| --- \| --- \| --- \| --- \| --- \| --- \| --- \| --- \| --- \| --- \| --- \| \| bits  5.9 \| \| \| \| \| \| \| \| \| \| \| \| \| \| \| \| \| \| \| \| \| \| \| \| \| \| \| \| \| \| \| 5.3 \| \| \| \| \| \| \| \| \| \| \| \| \| \| \| \| \| \| \| \| \| \| \| \| \| \| \| \| \| \| \| 4.7 \| \| \| \| \| \| \| \| \| \| \| \| \| \| \| \| \| \| \| \| \| \| \| \| \| \| \| \| \| \| \| 4.1 \| \| \| \| \| \| \| \| \| \| \| \| \| \| \| \| \| \| \| \| \| \| \| \| \| \| \| \| \| \| \| Information  3.5 \| \| \| \| \| \| \| \| \| \| \| \| \| \| \| \| \| \| \| \| \| \| \| \| \| \| \| \| \| \| \| content  2.9 \| \| \| \| \| \| \| \| \| \| \| \| \| \| \| \| \| \| \| \| \| \| \| \| \| \| \| \| \| \| \| (53.9 bits) 2.3 \| \| \| \| \| \| \| \| \| \| \| \| \| \| \| \| \| \| \| \| \| \| \| \| \| \| \| \| \| \| \| 1.8 \| \| \| \| \| \| \| \| \| \| \| \| \| \| \| \| \| \| \| \| \| \| \| \| \| \| \| \| \| \| \| 1.2 \| \| \| \| \| \| \| \| \| \| \| \| \| \| \| \| \| \| \| \| \| \| \| \| \| \| \| \| \| \| \| 0.6 \| \| \| \| \| \| \| \| \| \| \| \| \| \| \| \| \| \| \| \| \| \| \| \| \| \| \| \| \| \| \| 0.0 --- \| \| \| \| \| \| \| \| \| \| \| \| \| .\|  \|  \|  \|  \|  \|  \|  \|  \|  \|  \|  \|  \|  \|  \|  \|  \|  \|  \|  \|  \|  \|  \|  \|  \|  \|  \|  \|  \|  \|  \| \| --- \| --- \| --- \| --- \| --- \| --- \| --- \| --- \| --- \| --- \| --- \| --- \| --- \| --- \| --- \| --- \| --- \| --- \| --- \| --- \| --- \| --- \| --- \| --- \| --- \| --- \| --- \| --- \| --- \| --- \| \| Multilevel  **P** **V** **D** **N** **F** **D** **I** **S** **K** **F** **A** **G** **T** **W** **H** **E** **I** **A** **K** \| \| \| \| \| \| \| \| \| \| \| \| \| \| \| \| \| \| \| \| \| \| \| \| \| \| \| \| \| \| \| consensus  T G E D L N K A A I H A Y A L I L \| \| \| \| \| \| \| \| \| \| \| \| \| \| \| \| \| \| \| \| \| \| \| \| \| \| \| \| \| \| \| sequence  V K K G L E N V N E W S M M \| \| \| \| \| \| \| \| \| \| \| \| \| \| \| \| \| \| \| \| \| \| \| \| \| \| \| \| \| \| \| M N V Q R Y K T V \| \| \| \| \| \| \| \| \| \| \| \| \| \| \| \| \| \| \| \| \| \| \| \| \| \| \| \| \| \| \| R W \| \| \| \| \| \| \| \| \| \| \| \| \| \| \| \| \| \| \| \| \| \| \| \| \| \| \| \| \| \| \| .\| NAME START P-VALUE SITES \| \| \| \| \| \| \| \| \| \| \| \| \| \| \| \| \| \| \| \| \| \| \| \| \| \| \| \| \| \| \| \| --- \| --- \| --- \| --- \| --- \| --- \| --- \| --- \| --- \| --- \| --- \| --- \| --- \| --- \| --- \| --- \| --- \| --- \| --- \| --- \| --- \| --- \| --- \| --- \| --- \| --- \| --- \| --- \| --- \| --- \| --- \| \| \| |  |  |  |  |  |  |  |

 \| \| \| \| \| \| \| \| \| \| \| \| \| \| \| \| \| \| \| \| \| \| \| \| \| \| \| \| \| \| \| \| \| \| \| --- \| --- \| --- \| --- \| --- \| --- \| --- \| --- \| --- \| --- \| --- \| --- \| --- \| --- \| --- \| --- \| --- \| --- \| --- \| --- \| --- \| --- \| --- \| --- \| --- \| --- \| --- \| --- \| --- \| --- \| --- \| --- \| --- \| \| ICYA\_MANSE 14 1.43e-18 FYPGYCPDVK **P **V **N **D **F **D **L **S **A **F **A **G **A **W **H **E **I **A **K LPLENENQGK \| \| \|** \|** \|** \|** \|** \|** \|** \|** \|** \|** \|** \|** \|** \|** \|** \|** \|** \|** \|** \| \| \| \| \| \| \| \| \| \| \| \| \| BBP\_PIEBR 13 9.75e-18 YHDGACPEVK **P **V **D **N **F **D **W **S **N **Y **H **G **K **W **W **E **V **A **K YPNSVEKYGK \| \| \|** \|** \|** \|** \|** \|** \|** \|** \|** \|** \|** \|** \|** \|** \|** \|** \|** \|** \|** \| \| \| \| \| \| \| \| \| \| \| \| \| RETB\_BOVIN 11 1.99e-16 ERDCRVSSFR **V **K **E **N **F **D **K **A **R **F **A **G **T **W **Y **A **M **A **K KDPEGLFLQD \| \| \|** \|** \|** \|** \|** \|** \|** \|** \|** \|** \|** \|** \|** \|** \|** \|** \|** \|** \|** \| \| \| \| \| \| \| \| \| \| \| \| \| LACB\_BOVIN 22 9.62e-15 CGAQALIVTQ **T **M **K **G **L **D **I **Q **K **V **A **G **T **W **Y **S **L **A **M AASDISLLDA \| \| \|** \|** \|** \|** \|** \|** \|** \|** \|** \|** \|** \|** \|** \|** \|** \|** \|** \|** \|** \| \| \| \| \| \| \| \| \| \| \| \| \| MUP2\_MOUSE 24 4.53e-14 VCVHAEEASS **T **G **R **N **F **N **V **E **K **I **N **G **E **W **H **T **I **I **L ASDKREKIED \| \| \|** \|** \|** \|** \|** \|** \|** \|** \|** \|** \|** \|** \|** \|** \|** \|** \|** \|** \|** \| \| \| \| \| \| \| \| \| \| \| \| --- Motif 1 block diagrams --- Name Lowest p-value    Motifs ICYA\_MANSE 1.4e-18 --- 1 --- BBP\_PIEBR 9.7e-18 --- 1 --- RETB\_BOVIN 2e-16 --- 1 --- LACB\_BOVIN 9.6e-15 --- 1 --- MUP2\_MOUSE 4.5e-14 --- 1 --- SCALE \| \| \| \| \| \| \| \| 1 25 50 75 100 125 150 175 \| --- Motif 1 in BLOCKS format --- ``` BL MOTIF 1 width=19 seqs=5 ICYA_MANSE ( 14) PVNDFDLSAFAGAWHEIAK 1 BBP_PIEBR ( 13) PVDNFDWSNYHGKWWEVAK 1 RETB_BOVIN ( 11) VKENFDKARFAGTWYAMAK 1 LACB_BOVIN ( 22) TMKGLDIQKVAGTWYSLAM 1 MUP2_MOUSE ( 24) TGRNFNVEKINGEWHTIIL 1 // ``` --- Motif 1 position-specific scoring matrix --- ``` log-odds matrix: alength= 20 w= 19 n= 813 bayes= 7.33628 E= 8.9e-006 -93 -241 -296 -256 -247 -224 -228 -113 -258 -190 -106 -229 361 -141 -151 -73 242 71 -330 -325 -63 -187 -232 -163 -140 75 -134 22 37 -77 241 -166 -172 -51 -40 -100 -53 221 -226 -208 -71 -310 103 103 -254 -142 -60 -205 81 -207 -94 124 -126 52 201 -34 -50 -195 -285 -234 -242 -375 92 -196 -382 91 -98 -352 -243 -408 -311 349 -242 -122 -182 -80 -157 -389 -397 -351 -318 -290 -467 -456 425 -423 -363 -172 -484 -47 -115 -430 -331 -373 -362 -296 -352 -245 -202 -103 -301 -418 363 -117 -408 -310 -244 -367 -403 -415 -323 -21 -370 -260 -284 -260 -313 -381 -414 -410 -107 -180 -350 -287 -106 -249 -200 251 -5 95 46 -249 -221 -152 -148 -153 -77 137 217 -191 96 -298 -78 114 -258 -152 -75 -202 -65 -209 -96 -73 -125 216 7 200 -40 -190 -293 -244 64 -339 -187 -114 -314 -206 -99 -238 222 -242 -133 101 -185 18 221 -102 -106 -240 -310 -282 -180 -226 -378 -340 342 -310 -122 98 -351 -88 -39 -305 -258 -227 -212 -200 -176 43 -146 170 273 -215 -127 -140 -272 -123 196 -223 -154 -247 -141 118 -194 -56 -71 -37 -96 -174 -321 -276 -198 -392 -300 -366 -445 397 -328 -418 -373 -473 -335 -252 -325 -311 -239 -220 -319 -402 -398 -452 89 -295 -91 104 -246 -153 -67 -184 77 -196 -82 -70 -123 52 29 -26 223 -176 -280 -234 -388 -388 -438 -441 -134 -384 -353 -366 -445 -246 -240 -397 -380 -303 -275 -375 -362 -359 569 -207 -290 -318 -383 -376 74 -360 354 -251 -386 -230 -173 -259 -307 -176 -201 -252 -277 -283 285 292 106 -271 -97 173 -260 -150 -93 -197 -87 -214 -101 -85 -134 26 -13 137 156 -183 -299 -255 -245 -306 -430 -410 -197 -384 -360 356 -396 52 173 -359 -346 -288 -286 -287 -197 121 -336 -320 328 -140 -376 -329 -260 -140 -310 73 -352 -200 -114 -318 -308 -242 -222 -45 -138 -73 -332 -364 -191 -312 -315 -227 -234 -288 -184 -127 276 37 218 -223 -256 -77 45 -195 -163 -176 -307 -303 ``` --- Motif 1 position-specific probability matrix --- ``` letter-probability matrix: alength= 20 w= 19 n= 813 E= 8.9e-006 0.037648 0.005501 0.008765 0.013021 0.007819 0.012183 0.005337 0.021760 0.014270 0.023208 0.008862 0.010843 0.397695 0.011040 0.010997 0.035380 0.255507 0.113151 0.001765 0.005249 0.046148 0.008025 0.013645 0.024875 0.016458 0.096406 0.010301 0.055614 0.110953 0.050677 0.097771 0.016767 0.009858 0.020610 0.023801 0.029318 0.033050 0.320302 0.003617 0.011804 0.043768 0.003408 0.139637 0.157518 0.007437 0.021402 0.017205 0.011549 0.150282 0.020596 0.009613 0.125451 0.013591 0.041992 0.126344 0.046274 0.033756 0.017918 0.002407 0.009853 0.013340 0.002176 0.128895 0.019792 0.003065 0.108033 0.013208 0.004162 0.015830 0.005120 0.002130 0.596897 0.006075 0.012544 0.008899 0.033645 0.016038 0.004677 0.001109 0.004364 0.007897 0.003917 0.002680 0.003261 0.825220 0.003049 0.002105 0.014496 0.002983 0.062366 0.008294 0.002695 0.003269 0.002197 0.002554 0.007506 0.004168 0.012667 0.004284 0.024393 0.008872 0.001618 0.843839 0.034226 0.002560 0.006677 0.004782 0.003736 0.005249 0.004866 0.001963 0.045950 0.002506 0.004824 0.004397 0.009659 0.005427 0.004957 0.000981 0.002912 0.034158 0.008398 0.006029 0.010539 0.020783 0.010237 0.006517 0.271739 0.082408 0.167783 0.025414 0.009464 0.007008 0.010200 0.011270 0.020324 0.027943 0.178674 0.077865 0.013246 0.139154 0.003705 0.039637 0.169215 0.007267 0.020045 0.015457 0.011745 0.054561 0.020311 0.009476 0.032113 0.013669 0.130645 0.033095 0.233639 0.036218 0.018570 0.002276 0.009201 0.111703 0.002786 0.018702 0.034797 0.004921 0.013734 0.013134 0.009141 0.399576 0.016168 0.007306 0.106564 0.009040 0.033103 0.145351 0.028873 0.022907 0.013104 0.002023 0.007067 0.020475 0.006103 0.004954 0.007296 0.465058 0.006699 0.011153 0.094150 0.007527 0.046967 0.014044 0.006431 0.005439 0.006065 0.007205 0.014673 0.014079 0.093289 0.006305 0.162088 0.474242 0.006597 0.028236 0.029163 0.006587 0.024524 0.100914 0.010144 0.029454 0.015659 0.006915 0.120434 0.008452 0.019893 0.019239 0.045115 0.024453 0.020764 0.001875 0.007340 0.018080 0.001931 0.008523 0.006103 0.001978 0.898946 0.002679 0.002630 0.006445 0.003277 0.001808 0.009277 0.003409 0.003397 0.006001 0.012759 0.005207 0.004284 0.001098 0.002167 0.132127 0.003792 0.036446 0.158523 0.007898 0.019837 0.016307 0.013317 0.146270 0.022232 0.010409 0.032682 0.013896 0.041836 0.038534 0.048888 0.224220 0.020422 0.002492 0.009872 0.004843 0.001992 0.003278 0.003609 0.017084 0.003997 0.002255 0.003771 0.003911 0.015805 0.003499 0.003387 0.002340 0.003593 0.004663 0.004356 0.003890 0.005749 0.896136 0.011843 0.009595 0.003235 0.004805 0.005679 0.072166 0.004720 0.302602 0.008347 0.005911 0.017649 0.005569 0.008799 0.003868 0.008653 0.007805 0.010215 0.006998 0.009772 0.125254 0.378358 0.149280 0.004467 0.034739 0.254975 0.007168 0.020366 0.013608 0.012130 0.046891 0.019644 0.009145 0.029472 0.012858 0.035034 0.028705 0.151252 0.140126 0.019446 0.002175 0.008519 0.013123 0.003519 0.003461 0.004471 0.011097 0.004013 0.002139 0.563637 0.005489 0.124417 0.061137 0.004401 0.002947 0.003962 0.004313 0.007984 0.012184 0.160598 0.001691 0.005416 0.696958 0.011057 0.005051 0.007864 0.007129 0.021780 0.003024 0.078866 0.007471 0.021665 0.008337 0.005850 0.003845 0.005473 0.006750 0.042869 0.018347 0.041929 0.001740 0.003994 0.018990 0.003357 0.007668 0.015921 0.008558 0.007816 0.007252 0.019764 0.578991 0.111958 0.083502 0.011287 0.005527 0.017179 0.043000 0.015133 0.015396 0.020540 0.002070 0.006090 ``` ``` Time 1.04 secs. ``` --- **P** **N** MOTIF 2     width = 20     sites = 5     llr = 195     E-value = 1.3e-004 --- Simplified A : : : : : : : 2 : : : : : : 4 : 2 : : : \| \| \| \| \| \| \|; pos.-specific C : : : : : : : : : : : : : : : : : 2 : 6 \| \| \| \| \| \| \|; probability D : 2 2 : : : : 4 : a : 2 : : : : : : : : \| \| \| \| \| \| \|; matrix E 4 : : : : : : : : : : 2 : : : : : : : 2 \| \| \| \| \| \| \|; F 2 : : 4 : : : : : : : : : 4 : : 2 : : : \| \| \| \| \| \| \|; G : : : : : : : : : : : : : : : : 2 : : : \| \| \| \| \| \| \|; H : : : 2 : : : : : : : : : : : : : 2 : : \| \| \| \| \| \| \|; I : : : : : 4 2 : : : : : : : 2 4 : : : 2 \| \| \| \| \| \| \|; K : : 2 : : : : 2 : : : 6 2 : : : : : : : \| \| \| \| \| \| \|; L : 2 : : 2 : 6 : : : : : : : 4 2 : : 2 : \| \| \| \| \| \| \|; M : : : : : : : : : : : : : : : 2 : : 2 : \| \| \| \| \| \| \|; N 4 6 : : 2 : : : : : 2 : 6 : : : 2 : 2 : \| \| \| \| \| \| \|; P : : : 2 : : 2 : : : : : : : : : : : : : \| \| \| \| \| \| \|; Q : : : : : : : : : : : : : : : : 2 : : : \| \| \| \| \| \| \|; R : : : : : : : : : : : : : : : : : : : : \| \| \| \| \| \| \|; S : : : : : : : 2 : : : : : : : : : : 2 : \| \| \| \| \| \| \|; T : : 2 : 2 : : : a : : : 2 : : : : : : : \| \| \| \| \| \| \|; V : : 4 2 : 6 : : : : : : : : : 2 : : : : \| \| \| \| \| \| \|; W : : : : 4 : : : : : : : : : : : : : : : \| \| \| \| \| \| \|; Y : : : : : : : : : : 8 : : 6 : : : 6 2 : \| \| \| \| \| \| \|; . bits 5.9 \| \| \| \| \| \| \| 5.3 \| \| \| \| \| \| \| 4.7 \| \| \| \| \| \| \| 4.1 \| \| \| \| \| \| \| Information 3.5 \| \| \| \| \| \| \| content 2.9 \| \| \| \| \| \| \| (56.1 bits) 2.3 \| \| \| \| \| \| \| 1.8 \| \| \| \| \| \| \| 1.2 \| \| \| \| \| \| \| 0.6 \| \| \| \| \| \| \| 0.0 --- \| \| \| \| \| \| \| . Multilevel **E** **N** **V** **F** **W** **V** **L** **D** **T** **D** **Y** **K** **N** **Y** **A** **I** **A** **Y** **L** **C** \| \| \| \| \| \| \| consensus N D D H L I I A N D K F L L F C M E \| \| \| \| \| \| \| sequence F L K P N P K E T I M G H N I \| \| \| \| \| \| \| T V T S V N S \| \| \| \| \| \| \| Q Y \| \| \| \| \| \| \| . NAME START P-VALUE SITES ICYA\_MANSE 100 4.03e-19 MTFKFGQRVV **N **L **V **P **W **V **L **A **T **D **Y **K **N **Y **A **I **N **Y **N **C DYHPDKKAHS**************************************** BBP\_PIEBR 96 1.04e-18 HKLTYGGVTK **E **N **V **F **N **V **L **S **T **D **N **K **N **Y **I **I **G **Y **Y **C KYDEDKKGHQ**************************************** RETB\_BOVIN 101 2.40e-16 WGVASFLQKG **N **D **D **H **W **I **I **D **T **D **Y **E **T **F **A **V **Q **Y **S **C RLLNLDGTCA**************************************** MUP2\_MOUSE 105 6.24e-14 AGEYSVTYDG **F **N **T **F **T **I **P **K **T **D **Y **D **N **F **L **M **A **H **L **I NEKDGETFQL**************************************** LACB\_BOVIN 105 6.57e-13 PAVFKIDALN **E **N **K **V **L **V **L **D **T **D **Y **K **K **Y **L **L **F **C **M **E NSAEPEQSLA**************************************** \| \| \| \| \| \| \| --- Motif 2 block diagrams --- Name Lowest p-value    Motifs ICYA\_MANSE 4e-19 --- 2 --- BBP\_PIEBR 1e-18 --- 2 --- RETB\_BOVIN 2.4e-16 --- 2 --- MUP2\_MOUSE 6.2e-14 --- 2 --- LACB\_BOVIN 6.6e-13 --- 2 --- SCALE \| \| \| \| \| \| \| \| 1 25 50 75 100 125 150 175 \| --- Motif 2 in BLOCKS format --- ``` BL MOTIF 2 width=20 seqs=5 ICYA_MANSE ( 100) NLVPWVLATDYKNYAINYNC 1 BBP_PIEBR ( 96) ENVFNVLSTDNKNYIIGYYC 1 RETB_BOVIN ( 101) NDDHWIIDTDYETFAVQYSC 1 MUP2_MOUSE ( 105) FNTFTIPKTDYDNFLMAHLI 1 LACB_BOVIN ( 105) ENKVLVLDTDYKKYLLFCME 1 // ``` --- Motif 2 position-specific scoring matrix --- ``` log-odds matrix: alength= 20 w= 20 n= 808 bayes= 7.32733 E= 1.3e-004 -116 -324 -64 181 136 -144 -82 -221 -110 -238 -136 235 -162 0 -39 -57 -86 -222 -289 -199 -264 -361 -4 -243 -347 -231 -89 -277 -263 -94 -248 377 -263 -136 -183 -110 -174 -332 -348 -353 -66 -251 55 -62 -218 -174 -92 -65 47 -162 -65 -95 -142 12 -3 -58 128 212 -277 -241 -126 -208 -290 -239 294 -248 179 -55 -244 -93 -23 -219 151 -123 -126 -139 -115 66 -140 111 -163 -256 -286 -240 -117 -258 -187 -104 -233 -2 -61 22 -239 -123 -124 -153 70 -128 496 -172 -187 -259 -460 -433 -253 -411 -393 294 -445 -100 -72 -409 -350 -346 -317 -333 -179 284 -423 -403 -241 -288 -462 -384 -125 -379 -303 115 -384 294 55 -375 98 -219 -226 -288 -207 -90 -286 -303 87 -310 199 -26 -267 -139 -74 -219 69 -226 -114 -38 -135 29 -2 118 -60 -209 -302 -248 -207 -284 -375 -406 -362 -318 -321 -234 -360 -351 -189 -221 -300 -234 -236 -21 413 -235 -394 -430 -314 -425 368 -126 -416 -331 -261 -375 -424 -423 -332 -101 -381 -278 -298 -284 -333 -389 -419 -420 -225 -294 -274 -298 79 -283 -38 -226 -307 -220 -157 120 -267 -192 -179 -191 -229 -254 -84 364 -140 -422 109 152 -378 -212 -152 -292 227 -299 -190 -123 -188 11 -34 -125 -141 -275 -395 -345 -219 -352 -147 -205 -340 -210 -87 -272 -8 -326 -217 360 -237 -84 -81 -94 66 -308 -341 -333 -252 -295 -377 -356 284 -344 -34 -222 -366 -206 -151 -294 -288 -226 -210 -233 -263 -256 -55 339 187 -211 -422 -353 -120 -290 -268 186 -351 201 34 -320 -273 -212 -215 -199 -130 -4 -258 -258 -245 -306 -430 -410 -197 -384 -360 356 -396 52 173 -359 -346 -288 -286 -287 -197 121 -336 -320 101 -261 -102 -53 108 107 -47 -151 -62 -172 -64 119 -120 207 16 -25 -39 -150 -255 -163 -159 113 -291 -265 139 -272 185 -163 -283 -165 -93 -236 -215 -167 -141 -152 -187 -189 -63 335 -70 -186 -203 -142 -27 -188 -50 -16 -146 73 243 97 -158 -34 -48 101 -41 -46 -169 156 -296 492 -486 -201 -375 -433 -397 -94 -465 -327 -236 -427 -408 -348 -325 -347 -285 -273 -475 -467 ``` --- Motif 2 position-specific probability matrix --- ``` letter-probability matrix: alength= 20 w= 20 n= 808 E= 1.3e-004 0.032008 0.003091 0.043880 0.270369 0.111568 0.021231 0.014687 0.010339 0.039904 0.016638 0.007179 0.270043 0.010552 0.029256 0.023973 0.039284 0.026211 0.014867 0.002331 0.012589 0.011450 0.002401 0.066534 0.014318 0.003909 0.011552 0.014042 0.006973 0.013808 0.045272 0.003312 0.726528 0.005234 0.011368 0.008820 0.027339 0.014303 0.006960 0.001554 0.004324 0.045251 0.005136 0.099769 0.049888 0.009538 0.017240 0.013704 0.030460 0.118766 0.028112 0.011727 0.027391 0.012135 0.031892 0.030823 0.039152 0.115493 0.301591 0.002541 0.009392 0.029934 0.006911 0.009133 0.014670 0.332829 0.010301 0.090048 0.032519 0.015765 0.045539 0.015711 0.011656 0.092276 0.012458 0.013088 0.022310 0.021474 0.109455 0.006588 0.107334 0.023030 0.004946 0.009422 0.014560 0.019318 0.009618 0.007125 0.023162 0.017027 0.085376 0.012045 0.061630 0.006208 0.012490 0.013307 0.020257 0.077420 0.028644 0.539262 0.015151 0.019524 0.004853 0.002817 0.003837 0.007481 0.003333 0.001707 0.365109 0.003926 0.043264 0.011190 0.003119 0.002882 0.002667 0.003484 0.005829 0.013773 0.497223 0.000923 0.003058 0.013417 0.003965 0.002769 0.005369 0.018159 0.004139 0.003187 0.105761 0.005997 0.664352 0.027015 0.003938 0.064037 0.006423 0.006558 0.007970 0.011357 0.037092 0.002389 0.006106 0.130922 0.003407 0.270564 0.064021 0.006814 0.021865 0.015590 0.010435 0.137674 0.018102 0.008363 0.040899 0.012728 0.035797 0.031068 0.132991 0.031416 0.016267 0.002144 0.008934 0.017077 0.004075 0.005057 0.004623 0.003535 0.006314 0.002802 0.009391 0.007043 0.007620 0.004986 0.011481 0.004052 0.005781 0.006120 0.050675 0.832113 0.013602 0.001127 0.002525 0.008139 0.001535 0.873188 0.032034 0.002430 0.005774 0.004248 0.003537 0.004519 0.004622 0.001844 0.026334 0.002312 0.004257 0.003983 0.008174 0.004732 0.004687 0.000947 0.002703 0.014990 0.003804 0.010209 0.009730 0.074757 0.008093 0.020011 0.009966 0.010205 0.018869 0.006196 0.122202 0.005112 0.007713 0.009088 0.015562 0.009739 0.011934 0.009679 0.622142 0.027180 0.001567 0.145409 0.220364 0.003145 0.013192 0.009039 0.006306 0.411729 0.010929 0.004942 0.022649 0.008858 0.031479 0.024750 0.024543 0.017955 0.010291 0.001120 0.004554 0.015679 0.002549 0.024718 0.018622 0.004114 0.013434 0.014186 0.007243 0.080917 0.009062 0.004102 0.644220 0.006273 0.016301 0.017863 0.030405 0.075495 0.008221 0.001627 0.004969 0.012490 0.003782 0.004999 0.006533 0.311220 0.005292 0.020581 0.010241 0.006786 0.020735 0.006474 0.006937 0.004429 0.006107 0.007312 0.011667 0.007686 0.011774 0.011802 0.523153 0.261687 0.006758 0.003650 0.006646 0.018855 0.007670 0.004061 0.173482 0.007529 0.349203 0.023390 0.005783 0.004898 0.006730 0.007085 0.014687 0.019305 0.067370 0.002891 0.008320 0.013123 0.003519 0.003461 0.004471 0.011097 0.004013 0.002139 0.563637 0.005489 0.124417 0.061137 0.004401 0.002947 0.003962 0.004313 0.007984 0.012184 0.160598 0.001691 0.005416 0.143897 0.004796 0.033739 0.053310 0.091558 0.120306 0.018800 0.016682 0.055672 0.026293 0.011807 0.121340 0.014186 0.123220 0.035201 0.049209 0.036293 0.024574 0.002968 0.016149 0.023811 0.063840 0.009090 0.012295 0.113437 0.008693 0.094037 0.015423 0.012067 0.027627 0.009638 0.010374 0.007336 0.009197 0.011843 0.020453 0.013020 0.018662 0.011229 0.507930 0.043881 0.008037 0.016679 0.028677 0.035909 0.015637 0.018373 0.042632 0.031185 0.143414 0.099065 0.103743 0.010902 0.023069 0.022578 0.117731 0.035868 0.050564 0.005357 0.146699 0.009178 0.885360 0.002350 0.019074 0.003228 0.002854 0.001658 0.024777 0.003400 0.009005 0.003589 0.002760 0.001926 0.002623 0.003308 0.005279 0.006605 0.010428 0.000645 0.001953 ``` ``` Time 1.47 secs. ``` --- **P** **N** MOTIF 3     width = 18     sites = 5     llr = 163     E-value = 1.2e+002 --- Simplified A : : : 2 2 : 2 2 : : : : : : : : : 2 \| \| \| \| \| \| \|; pos.-specific C : : : : : : : : : : : : : : : : : : \| \| \| \| \| \| \|; probability D : 2 2 2 : : : : : : : : 2 : : : : : \| \| \| \| \| \| \|; matrix E : : 2 : : : : : 4 : : : : 2 2 : 2 : \| \| \| \| \| \| \|; F 6 : : 2 : : : : : 2 : : : : : 4 : : \| \| \| \| \| \| \|; G : : : : : : : : : : : : : : : 6 : : \| \| \| \| \| \| \|; H 4 : : 2 : 2 : : : : 2 : : : : : 4 : \| \| \| \| \| \| \|; I : : 2 : 2 2 2 : : : : 2 : : : : : : \| \| \| \| \| \| \|; K : : 2 : : 2 : : : 4 : 4 : : 2 : : : \| \| \| \| \| \| \|; L : 4 : : 2 : : 8 : : : 2 : 8 : : : : \| \| \| \| \| \| \|; M : : : : : : : : : 2 : : : : : : : 2 \| \| \| \| \| \| \|; N : : : : 2 : : : : : : : : : 2 : 4 : \| \| \| \| \| \| \|; P : : : : : : : : 2 : : : : : : : : 2 \| \| \| \| \| \| \|; Q : 2 2 2 : : : : : : : : : : : : : : \| \| \| \| \| \| \|; R : : : : : : : : : 2 : : 2 : : : : : \| \| \| \| \| \| \|; S : 2 : : : : : : 4 : 8 : : : 2 : : : \| \| \| \| \| \| \|; T : : : : : : : : : : : : : : 2 : : 4 \| \| \| \| \| \| \|; V : : : : 2 : 6 : : : : 2 6 : : : : : \| \| \| \| \| \| \|; W : : : : : 4 : : : : : : : : : : : : \| \| \| \| \| \| \|; Y : : : : : : : : : : : : : : : : : : \| \| \| \| \| \| \|; . bits 5.9 \| \| \| \| \| \| \| 5.3 \| \| \| \| \| \| \| 4.7 \| \| \| \| \| \| \| 4.1 \| \| \| \| \| \| \| Information 3.5 \| \| \| \| \| \| \| content 2.9 \| \| \| \| \| \| \| (47.0 bits) 2.3 \| \| \| \| \| \| \| 1.8 \| \| \| \| \| \| \| 1.2 \| \| \| \| \| \| \| 0.6 \| \| \| \| \| \| \| 0.0 --- \| \| \| \| \| \| \| . Multilevel **F** **L** **D** **A** **A** **W** **V** **L** **E** **K** **S** **K** **V** **L** **E** **G** **H** **T** \| \| \| \| \| \| \| consensus H D E D I H A A S F H I D E K F N A \| \| \| \| \| \| \| sequence Q I F L I I P M L R N E M \| \| \| \| \| \| \| S K H N K R V S P \| \| \| \| \| \| \| Q Q V T \| \| \| \| \| \| \| . NAME START P-VALUE SITES MUP2\_MOUSE 59 1.43e-16 KIEDNGNFRL **F **L **E **Q **I **H **V **L **E **K **S **L **V **L **K **F **H **T VRDEECSELS************************************ ICYA\_MANSE 128 4.33e-16 NCDYHPDKKA **H **S **I **H **A **W **I **L **S **K **S **K **V **L **E **G **N **T KEVVDNVLKT************************************ BBP\_PIEBR 124 6.65e-16 YCKYDEDKKG **H **Q **D **F **V **W **V **L **S **R **S **K **V **L **T **G **E **A KTAVENYLIG************************************ RETB\_BOVIN 36 2.92e-11 AMAKKDPEGL **F **L **Q **D **N **I **V **A **E **F **S **V **D **E **N **G **H **M SATAKGRVRL************************************ LACB\_BOVIN 152 3.37e-11 PEVDDEALEK **F **D **K **A **L **K **A **L **P **M **H **I **R **L **S **F **N **P TQLEEQCHI************************************ \| \| \| \| \| \| \| --- Motif 3 block diagrams --- Name Lowest p-value    Motifs MUP2\_MOUSE 1.4e-16 --- 3 --- ICYA\_MANSE 4.3e-16 --- 3 --- BBP\_PIEBR 6.7e-16 --- 3 --- RETB\_BOVIN 2.9e-11 --- 3 --- LACB\_BOVIN 3.4e-11 --- 3 --- SCALE \| \| \| \| \| \| \| \| 1 25 50 75 100 125 150 175 \| --- Motif 3 in BLOCKS format --- ``` BL MOTIF 3 width=18 seqs=5 MUP2_MOUSE ( 59) FLEQIHVLEKSLVLKFHT 1 ICYA_MANSE ( 128) HSIHAWILSKSKVLEGNT 1 BBP_PIEBR ( 124) HQDFVWVLSRSKVLTGEA 1 RETB_BOVIN ( 36) FLQDNIVAEFSVDENGHM 1 LACB_BOVIN ( 152) FDKALKALPMHIRLSFNP 1 // ``` --- Motif 3 position-specific scoring matrix --- ``` log-odds matrix: alength= 20 w= 18 n= 818 bayes= 7.34518 E= 1.2e+002 -356 -356 -440 -445 360 -395 350 -258 -427 -226 -191 -316 -358 -249 -262 -312 -324 -305 -82 63 -69 -284 84 -24 -219 -147 -63 -149 -65 134 -62 -59 -123 214 12 116 -45 -158 -271 -221 -72 -328 98 131 -265 -163 -76 105 76 -211 -98 -82 -128 220 11 -49 -60 -195 -298 -247 71 -277 65 -47 99 -150 250 -173 -63 -180 -70 -56 -120 215 22 -34 -46 -170 -234 -51 68 -177 -342 -281 -121 -247 -203 236 -279 75 33 59 -220 -154 -152 -150 -75 170 -224 -212 -141 -265 -244 -175 -116 -232 175 98 42 -144 -64 -173 -207 -42 -4 -133 -112 -135 449 -110 11 -214 -387 -353 -251 -342 -291 134 -380 -154 -102 -357 -264 -275 -224 -267 -119 327 -380 -402 11 -293 -461 -384 -138 -354 -314 -36 -386 309 44 -379 -291 -224 -230 -268 -216 -115 -300 -319 -96 -337 -65 195 -316 -171 -125 -253 -117 -262 -155 -91 215 10 -54 207 -85 -236 -355 -302 -132 -302 -217 -136 105 -220 -107 -166 221 -190 230 -143 -192 3 217 -114 -105 -183 -281 -218 -137 -246 -231 -280 -303 -216 152 -307 -260 -336 -220 -124 -230 -176 -152 352 81 -324 -355 -310 -122 -196 -348 -279 -130 -265 -212 221 130 100 24 -255 -236 -148 -130 -169 -93 145 -242 -229 -82 -224 -28 -176 -246 -260 -191 13 -196 -173 -101 -213 -210 -112 92 -171 -94 311 -336 -333 -257 -317 -366 -34 -145 -376 -293 -49 -335 311 44 -349 -283 -186 -199 -286 -226 -133 -303 -318 -61 -290 -84 89 -239 -134 -52 -191 75 -197 -82 123 -117 53 29 134 145 -184 -273 -222 -188 -343 -328 -380 240 348 -270 -306 -387 -326 -253 -265 -315 -287 -255 -208 -268 -315 -281 -187 -179 -348 -75 74 -294 -152 363 -312 -163 -307 -203 241 -200 -16 -67 -77 -127 -308 -328 -217 94 -202 -240 -183 -193 -189 -164 -82 -186 -143 244 -162 213 -72 -88 -16 269 -93 -268 -252 ``` --- Motif 3 position-specific probability matrix --- ``` letter-probability matrix: alength= 20 w= 18 n= 818 E= 1.2e+002 0.006081 0.002485 0.003227 0.003521 0.524935 0.003723 0.294451 0.007980 0.004452 0.018070 0.004884 0.005929 0.002720 0.005205 0.005113 0.006744 0.005063 0.008392 0.009807 0.077217 0.044364 0.004099 0.122591 0.065047 0.009508 0.020693 0.016794 0.016958 0.054726 0.219610 0.011961 0.035373 0.013850 0.128524 0.034140 0.130314 0.034809 0.023236 0.002656 0.010747 0.043525 0.003007 0.135076 0.190510 0.006881 0.018595 0.015392 0.098740 0.144497 0.020033 0.009351 0.030171 0.013395 0.134647 0.034017 0.041547 0.031465 0.017979 0.002195 0.008978 0.116613 0.004303 0.106973 0.055462 0.086279 0.020274 0.147413 0.014401 0.055315 0.024957 0.011360 0.035936 0.014112 0.129452 0.036516 0.046294 0.034558 0.021410 0.003426 0.034946 0.114267 0.008562 0.006373 0.010972 0.018716 0.010393 0.006385 0.245069 0.012418 0.145730 0.023072 0.080132 0.007067 0.010046 0.010978 0.020619 0.028362 0.225686 0.003660 0.011494 0.026978 0.004647 0.012562 0.022916 0.019425 0.011461 0.087303 0.093998 0.114768 0.032011 0.011827 0.016012 0.007725 0.021901 0.030487 0.023252 0.021909 0.027229 0.390373 0.023216 0.076974 0.006631 0.004672 0.006673 0.007600 0.005349 0.003455 0.120557 0.006143 0.029791 0.009063 0.004482 0.005218 0.004337 0.006660 0.009194 0.020826 0.668049 0.001248 0.003077 0.077273 0.003833 0.002798 0.005358 0.016607 0.004930 0.002954 0.037170 0.005887 0.738688 0.025054 0.003828 0.004338 0.006203 0.006400 0.009099 0.010656 0.031297 0.002160 0.005466 0.036647 0.002835 0.043369 0.297987 0.004857 0.017507 0.010937 0.008268 0.037961 0.014088 0.006306 0.028278 0.143873 0.031430 0.021590 0.246399 0.026533 0.013495 0.001485 0.006157 0.028576 0.003595 0.015146 0.029979 0.089976 0.012532 0.012350 0.015081 0.396978 0.023178 0.090574 0.019670 0.008613 0.029777 0.141343 0.026547 0.023098 0.019534 0.002475 0.010978 0.027726 0.005313 0.013740 0.011015 0.005303 0.012810 0.074620 0.005664 0.014139 0.008466 0.004008 0.022471 0.006616 0.008663 0.010919 0.670344 0.083569 0.007326 0.001484 0.005803 0.030695 0.007530 0.006117 0.011131 0.017578 0.009122 0.005993 0.220376 0.211453 0.173241 0.021747 0.009090 0.006333 0.010507 0.012760 0.018124 0.025050 0.189682 0.003250 0.010220 0.040589 0.006172 0.056063 0.022680 0.007866 0.009464 0.006932 0.052284 0.022058 0.026167 0.009146 0.012141 0.007608 0.013420 0.059535 0.017886 0.024791 0.598543 0.001684 0.004972 0.012022 0.003248 0.005396 0.060794 0.015895 0.004251 0.003405 0.033952 0.008400 0.749174 0.025007 0.004710 0.004572 0.008073 0.007912 0.008044 0.009937 0.027586 0.002116 0.005505 0.046781 0.003907 0.038043 0.142536 0.008253 0.022677 0.018086 0.012647 0.143644 0.022067 0.010407 0.124330 0.014421 0.042345 0.038480 0.148474 0.130260 0.019378 0.002605 0.010661 0.019455 0.002715 0.007040 0.005518 0.229289 0.641445 0.004005 0.005735 0.005871 0.009036 0.003191 0.008432 0.003650 0.003992 0.005379 0.013862 0.007456 0.007802 0.002479 0.013647 0.020610 0.002616 0.040582 0.128857 0.005659 0.020053 0.321706 0.005492 0.027590 0.010317 0.004518 0.282932 0.008106 0.026192 0.019685 0.034217 0.019793 0.008215 0.001781 0.011078 0.137648 0.007194 0.012935 0.021570 0.011374 0.015531 0.008361 0.026917 0.023613 0.032221 0.100110 0.017268 0.142155 0.017699 0.017053 0.052513 0.308143 0.036319 0.002706 0.008668 ``` ``` Time 1.83 secs. ``` --- **P** **N** SUMMARY OF MOTIFS --- --- Combined block diagrams: non-overlapping sites with p-value < 0.0001 --- Name Combined p-value    Motifs ICYA\_MANSE 6.82e-42 --- 1 --- 2 --- 3 --- LACB\_BOVIN 2.15e-27 --- 1 --- 2 --- 3 --- BBP\_PIEBR 1.29e-40 --- 1 --- 2 --- 3 --- RETB\_BOVIN 2.11e-32 --- 1 --- 3 --- 2 --- MUP2\_MOUSE 5.96e-33 --- 1 --- 3 --- 2 --- SCALE \| \| \| \| \| \| \| \| 1 25 50 75 100 125 150 175 \| --- Stopped because nmotifs = 3 reached. --- ``` CPU: nbcr2 ``` --- EXPLANATION OF MEME RESULTS --- The MEME results consist of: The **version** of MEME and the date it was released. The **reference** to cite if you use MEME in your research. A description of the **sequences** you submitted (the "training set") showing the name, "weight" and length of each sequence. The **command line summary** detailing the parameters with which you ran MEME. Information on each of the **motifs** MEME discovered, including: 1. A summary line showing the width, number of occurrences, log likelihood ratio and statistical significance of the motif.- A simplified position-specific probability matrix.- A diagram showing the degree of conservation at each motif position.- A multilevel consensus sequence showing the most conserved letter(s) at each motif position.- The occurrences of the motif sorted by *p*-value and aligned with each other.- Block diagrams of the occurrences of the motif within each sequence in the training set.- The motif in BLOCKS or FASTA format.- A position-specific scoring matrix (PSSM) for use by the MAST database search program.- The position specific probability matrix (PSPM) describing the motif. A **summary of motifs** showing an optimized (non-overlapping) tiling of all of the motifs onto each of the sequences in the training set. The reason why MEME stopped and the name of the CPU on which it ran. This **explanation** of how to interpret MEME results. MOTIFS For each motif that it discovers in the training set, MEME prints the following information: - Summary Line This line gives the width (`width'), number of occurrences in the training set (`sites'), log likelihood ratio (`llr') and *E*-value of the motif. Each motif describes a pattern of a fixed width--no gaps are allowed in MEME motifs. MEME numbers the motifs consecutively from one as it finds them. MEME usually finds the most statistically significant (low *E*-value) motifs first. The statistical significance of a motif is based on its log likelihood ratio, its width and number of occurrences, the background letter frequencies (given in the command line summary), and the size of the training set. The *E*-value is an estimate of the expected number of motifs with the given log likelihood ratio (or higher), and with the same width and number of occurrences, that one would find in a similarly sized set of random sequences. (In random sequences each position is independent with letters chosen according to the background letter frequencies.) The log likelihood ratio is the logarithm of the ratio of the probability of the occurrences of the motif given the motif model (likelihood given the motif) versus their probability given the background model (likelihood given the null model). (Normally the background model is a 0-order Markov model using the background letter frequencies, but higher order Markov models may be specified via the **-bfile** option to MEME.) Clicking on the **buttons** to the left of the motif summary line takes you to the previous motif (P) or next motif (N). - Simplified Position-Specific Probability Matrix MEME motifs are represented by position-specific probability matrices that specify the probability of each possible letter appearing at each possible position in an occurrence of the motif. In order to make it easier to see which letters are most likely in each of the columns of the motif, the simplified motif shows the letter probabilities multiplied by 10 rounded to the nearest integer. Zeros are replaced by ":" (the colon) for readability. - Information Content Diagram The information content diagram provides an idea of which positions in the motif are most highly conserved. Each column (position) in a motif can be characterized by the amount of information it contains (measured in bits). Highly conserved positions in the motif have high information; positions where all letters are equally likely have low information. (The information content is relative to the background letter frequencies which are given in the command line summary section.) The diagram is printed so that each column lines up with the same column in the simplified position-specific probability matrix above it. Columns in the information content diagram are colored according to the majority category of the letters occurring in that column of the alignment. If no letter category has frequency above 0.5, the column in the diagram is colored black. For DNA sequences, the letter categories contain one letter each. For proteins, the categories are based on the biochemical properties of the various amino acids. The categories and their colors are: NUCLEIC ACIDS \| COLOR; A \| RED; C \| BLUE; G \| ORANGE; T \| GREEN AMINO ACIDS \| COLOR; ACFILVM \| BLUE; NQST \| GREEN; DE \| MAGENTA; KR \| RED; H \| PINK; G \| ORANGE; P \| YELLOW; Y \| TURQUOISE Summing the information content for each position in the motif gives the total information content of the motif (shown in parentheses to the left of the diagram). The total information content is approximately equal to the log likelihood ratio divided by the number of occurrences times ln(2). The total information content gives a measure of the usefulness of the motif for database searches. For a motif to be useful for database searches, it must as a rule contain at least log\_2(N) bits of information where N is the number of sequences in the database being searched. For example, to effectively search a database containing 100,000 sequences for occurrences of a *single* motif, the motif should have an IC of at least 16.6 bits. Motifs with lower information content are still useful when a family of sequences shares more than one motif since they can be combined in *multiple* motif searches (using MAST). - Multilevel Consensus Sequence The multilevel consensus sequence corresponding to the motif is an aid in remembering and understanding the motif. It is calculated from the motif position-specific probability matrix as follows. Separately for each column of the motif, the letters in the alphabet are sorted in decreasing order by the probability with which they are expected to occur in that position of motif occurrences. The sorted letters are then printed vertically with the most probable letter on top. Only letters with probabilities of 0.2 or higher at that position in the motif are printed. As an example, the multilevel consensus sequence of motif 1 in the sample output is: ``` Multilevel TTATGTGAACGACGTCACACT consensus AA T A G A GA AA sequence T C TT T ``` This multilevel consensus sequence says several things about the motif. First, the most likely form of the motif can be read from the top line as TTATGTGAACGACGTCACACT. Second, that only letter A has probability more than 0.2 in position 3 of the motif, both T and A have probability greater than 0.2 in position 1, etc. Third, a *rough approximation* of the motif can be made by converting the multilevel consensus sequence into the Prosite signature [TA]-[TA]-A-T-[GT]-[T]-[GA]-A-[AGT]-C-[GAC]-A-[CGT]-[GAT]-T-C-A-C-A-[CAT]-[TA] . - Occurrences of the Motif MEME displays the occurrences (sites) of the motif in the training set. The sites are shown aligned with each other, and the ten sequence positions preceding and following each site are also shown. Each site is identified by the name of the sequence where it occurs, the strand (if both strands of DNA sequences are being used), and the position in the sequence where the site begins. When the DNA strand is specified, `+' means the sequence in the training set, and `-' means the reverse complement of the training set sequence. (For `-' strands, the `start' position is actually the position on the **positive** strand where the site ends.) The sites are listed in order of increasing statistical significance (*p*-value). The *p*-value of a site is computed from the the match score of the site with the position specific scoring matrix for the motif. The *p*-value gives the probability of a random string (generated from the background letter frequencies) having the same match score or higher. (This is referred to as the **position *p*-value** by the MAST algorithm.) - Block Diagrams of Motif Occurrences The occurrences of the motif in the training set sequences are shown with MAST-style block diagrams. One diagram is printed for each sequence showing all the occurrences of the motif in that sequence. The sequences are sorted by the **lowest** *p*-value among all occurrences of the motif in a given sequence. (The *p*-value of an occurrence is the probability of a single random subsequence the length of the motif, generated according to the 0-order background model, having a score at least as high as the score of the occurrence.) When the DNA strand is specified, `+' means the motif appears from left to right on the sequence, and `-' means the motif appears from right to left on the complementary strand. A sequence position scale is shown at the end of each table of block diagrams. Very long sequences are shown with thick lines connecting the motifs and are **not** drawn to scale. - Motif in BLOCKS format or FASTA format For use with BLOCKS tools, MEME prints the occurrences of the motif in BLOCKS format. You can convert these blocks to PSSMs (position-specific scoring matrices), LOGOS (color representations of the motifs), phylogeny trees and search them against a database of other blocks by pasting everything from the "BL" line to the "//" line (inclusive) into the Multiple Alignment Processor. If you include the **-print\_fasta** switch on the command line, MEME prints the motif sites in FASTA format instead of BLOCKS format. - Position-Specific Scoring Matrix The position-specific scoring matrix corresponding to the motif is printed for use by database search programs such as MAST. This matrix is a log-odds matrix calculated by taking the log (base 2) of the ratio p/f at each position in the motif where p is the probability of a particular letter at that position in the motif, and f is the background frequency of the letter (given in the command line summary section.) This is the same matrix that is used above in computing the *p*-values of the occurrences of the motif in the Occurrences of the Motif and Block Diagrams of Motif Occurrences sections. The scoring matrix is printed "sideways"--columns correspond to the letters in the alphabet (in the same order as shown in the simplified motif) and rows corresponding to the positions of the motif, position one first. The scoring matrix is preceded by a line starting with "log-odds matrix:" and containing the length of the alphabet, width of the motif, number of characters in the training set and the scoring threshold used in the list of possible motif examples. - Position-Specific Probability Matrix The motif itself is a position-specific probability matrix giving, for each position in the pattern, the probabilities of each possible letter occurring there. The probability matrix is printed "sideways"--columns correspond to the letters in the alphabet (in the same order as shown in the simplified motif) and rows corresponding to the positions of the motif, position one first. The motif is preceded by a line starting with "letter-probability matrix:" and containing the length of the alphabet, width of the motif and number of characters in the training set. --- **Go to top** Meta-MEME program documentation Meta-MEME home |
